# Development of a handwashing with soap intervention in low-income settlements of Mombasa, Kenya

**DOI:** 10.1186/s41182-025-00842-1

**Published:** 2025-11-18

**Authors:** Sheillah N. Simiyu, Phylis J. Busienei, Naomi Njeri, Kelly K. Baker, Robert Dreibelbis

**Affiliations:** 1 African Population and Health Research Center, Nairobi, Kenya; 2 University at Buffalo State University of New York, Buffalo, NY USA; 3 London School of Hygiene and Tropical Medicine, London, UK

## Abstract

**Introduction:**

Interventions to improve handwashing with soap have shown mixed effects on behaviour which may be due to contextual differences in different settings. Low-income settings have complex socio-economic conditions which requires local contextual adaptation to support intervention adoption. Detailing the development of an intervention can inform other researchers and practitioners on best practices, and it enables replicability and scalability.

**Methods:**

This study adopted the Trials of Improved Practices (TIPs) approach and incorporated co-creation and co-design of interventions with stakeholders. A total of 56 participants were randomly selected and an initial survey was conducted. The development process entailed stakeholder engagements and educational activities. Educational activities were delivered through household-level visits and community dialogue sessions. Qualitative data were collected throughout the process using in-depth interviews. A survey was conducted after the educational activities to assess availability of handwashing facilities and handwashing with soap practices. Logistic regression was used to estimate the effect of independent variables on availability of handwashing facilities and on handwashing with soap, and McNemar’s test was used to evaluate if the interventions improved handwashing practices. Qualitative data were analysed thematically, and the findings explained the process and the effect of the interventions.

**Results:**

Initial survey results showed that 59% of handwashing facilities were not at a fixed location, and only 21% of respondents reported handwashing with soap. Households with a fixed handwashing facility had 5.3 times higher odds of handwashing with soap compared to households with mobile handwashing facilities (*P* = 0.02 CI 1.32–21.23). Participating households made improvements by designating handwashing facilities at the compound level and separate handwashing facilities at the household level. Access to fixed handwashing facilities increased from 10 to 77%, and reported handwashing with soap among respondents significantly increased from 21 to 64% after the education activities (McNemar's *X*^*2*^(1) = 12.46; *P* = 0.00). This improvement in handwashing was attributed to the educational visits and practical demonstrations and was motivated by improved hygiene conditions in the households.

**Implications:**

Households can improve reported handwashing with soap if they are provided with the necessary skills for making improvements. This approach could serve as a model for future public health initiatives aimed at improving hygiene practices in similar settings**.**

**Supplementary Information:**

The online version contains supplementary material available at 10.1186/s41182-025-00842-1.

## Introduction

Handwashing with soap is an effective public health measure that can reduce the risk of diarrheal diseases by up to 30% and that of respiratory infections by up to 17% [1, 2]. Various interventions have been implemented to increase uptake and adherence to handwashing with soap. These interventions used psycho-social theories, increased the community’s knowledge, or engaged the community in decision-making to address barriers to handwashing with soap [3]. A review of the effectiveness of these interventions in increasing uptake and adherence to handwashing with soap shows that interventions that increase the community’s knowledge are generally effective in increasing uptake of handwashing with soap, while those that adopt a psycho-social approach are associated with sustained adherence to handwashing with soap [3]. Yet, despite these general trends, several studies on handwashing with soap have reported mixed results on uptake and adherence of handwashing with soap; [4–8] which may be due to the differences in contexts (e.g. urban, rural or humanitarian settings) where the approaches were implemented.

Differences in contexts require context specific handwashing with soap interventions. As such, a comprehensive assessment and understanding of the community should first be conducted so that interventions that are developed are specific to and acceptable in the community [9]. Such an assessment entails understanding the enablers and barriers to the adoption of positive practices. Factors such as beliefs, knowledge, costs, and access to handwashing facilities and resources within a community may be barriers or enablers to uptake of handwashing with soap practices and they determine the most appropriate interventions that increase uptake and adherence [10, 11].

In Africa, urban and rural areas have different socio-economic and environmental contexts, calling for adequate understanding of these differences and which leads to implementation of appropriate interventions. Unlike rural residents, urban residents generally have higher levels of education, higher exposure to handwashing messaging, and higher access to handwashing facilities; which facilitate uptake of handwashing interventions [12]. However, studies from low-income urban areas indicate complex challenges of lack of reliable supply of water, inadequate hygiene facilities, low incomes, insecure land tenure, and social and political marginalization which affect uptake and adherence to handwashing interventions [13]. Development of handwashing interventions in such low-income settlements therefore requires thorough understanding of these challenges as well as opportunities for improvement.

Successful handwashing with soap interventions in low-income urban areas have incorporated provision of handwashing infrastructure, social approaches, and education [8, 14]. Yet, other factors such as collaboration and engagement with stakeholders during intervention development and implementation have also been singled out as factors that lead to success through enhanced social inclusion, development of contextually relevant interventions, and improved public health outcomes [15–19]. Detailing the development of these interventions is critical as it provides a reference for replicability and scalability, identifies best practices, and provides guidance for evaluation of the interventions [20].

Despite this importance, there remains a notable gap in describing the development of hygiene interventions tailored to urban settings in sub-Saharan Africa. While numerous studies have documented the design and implementation of handwashing interventions across schools, urban settings, and rural communities [21–24], such research within the African context is comparatively limited. Existing studies in Africa predominantly focus on handwashing interventions related to food hygiene and child health [25–27]. Moreover, studies specifically addressing the development of handwashing interventions in low-income urban areas in Africa are even fewer, and those that are available focus on certain aspects of intervention design or evaluation; for example, Sutherland et al. [28] describe the testing of an existing handwashing intervention rather than detailing the development process.

The objective of this paper, therefore, is to describe the process undertaken in developing a contextually appropriate intervention to promote handwashing with soap in a low-income urban setting in Africa. The development process entailed stakeholder engagement and research activities. We aim to explain activities conducted to develop the intervention as well as research activities that were conducted before and after developing and testing the intervention to assess the effect of the intervention on access to handwashing facilities and on handwashing with soap practices.

## Methods

### Study area

The study was conducted from September 2023 to June 2024 in Kisauni Sub-County of Mombasa city in Kenya. Mombasa city is in Mombasa County, and is the second-largest city in Kenya with an estimated population of 1.4 million people, 65% of whom live in low-income areas [29, 30]. Kisauni Sub-County has an estimated population of 300,000 people and is the largest and most populated Sub-County in Mombasa County [29]. The Sub-County has several low-income areas characterized by inadequate housing units, mud-walled or temporary housing structures, poor waste management, inadequate water supply and sanitation facilities, and poor road conditions [31]. Most of the houses are in compounds that have one common entrance and a common yard where the water point (often times a tap) and sanitation facilities are located. The settlements are covered by the Community Health Strategy system, which has communities divided into community units that are headed by a Community Health Promoter (CHP) who links the community members to primary health care services. After engagements with stakeholders, Machafuko community unit in Junda area which has approximately 3,000 households was selected for the study.

### Approach

The intervention development process combined engagement and education activities. Engagement activities comprised participatory discussions/meetings with stakeholders to facilitate collaborative input. Educational components were primarily delivered through household-level visits, supplemented by visual aids (e.g. posters) and community dialogue sessions all aimed at enhancing knowledge and practice of handwashing with soap. The development process was iterative, adopting principles of co-creation, co-design and co-production by involving the relevant stakeholders in identification, design and implementation of the interventions [17]. The Trials of Improved Practices (TIPs) approach [32] was adopted to refine and test the interventions with households. TIPs is a participatory approach often implemented after a formative phase to pilot-test and refine solutions/interventions before large scale implementation. Selected interventions are agreed upon and tested by participants, who then provide feedback that is used to further refine the interventions. TIPs uses a small sample size of 20–50 participants and lasts for a minimum of one week, depending on the behaviour being tested [32]. The approach requires flexibility and learning from the participants in order to fine tune the intervention that should resolve the public health problem [32].

**Fig. 1:**
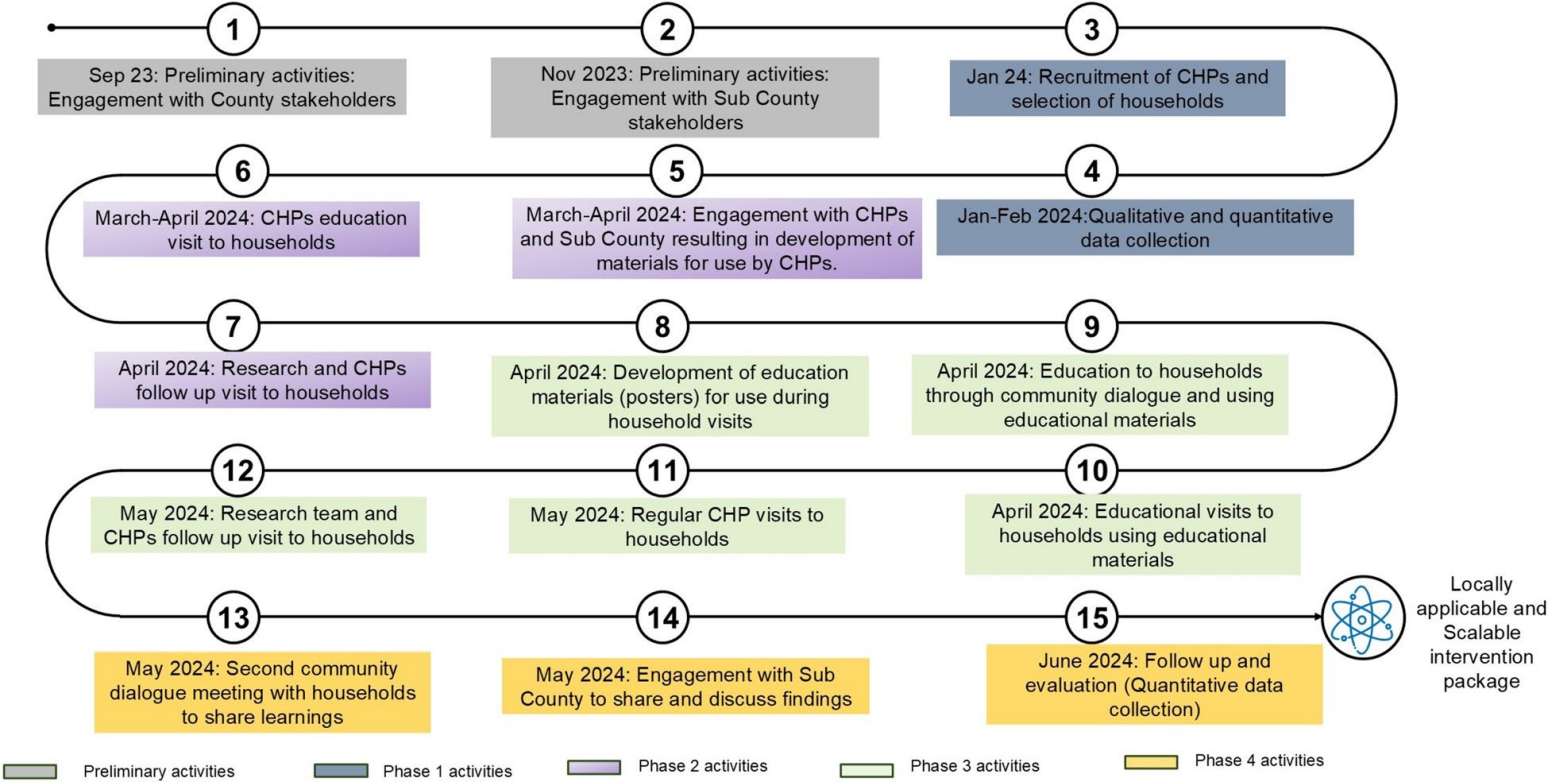
Flow of activities during intervention development process

Engagement and research activities were conducted prior to and throughout the intervention development process. A mixed-methods approach was employed for data collection. Quantitative data were collected through surveys administered to household heads before and after the educational visits and data were collected electronically through the survey CTO platform. Qualitative data were obtained through in-depth interviews with household respondents and group discussions with stakeholders during the development process. Due to the iterative nature of the intervention development process, findings from each phase informed subsequent activities, and thus, in- depth interviews guides were improved to follow up on previous activities. As such methods and activities will be presented with results to provide a comprehensive understanding of the intervention development process. An overall presentation of the flow of activities during the development process is presented in Fig. 1.

### Data *management* and analysis

Survey data were stored on a secure server after which it was downloaded to STATA (Stata Corp, V.15) for management and analysis. Analysis entailed running descriptive statistics to summarize the findings. Bivariate analysis (Pearson’s Chi-square test and logistic regression) was conducted to assess the relationship between variables. Significant associations in the Chi-square test were further assessed using logistic regression to estimate the effect of each independent variable on the outcome variables. McNemar’s test was used to test for differences in handwashing behaviour before and after the intervention. Statistical significance was declared at a *P*-value of ≤ 0.05.

Qualitative data were captured using voice recorders. The audio files were then transcribed into Microsoft word and translated to English. Analysis entailed a thematic approach where codes and themes were identified beforehand. The transcripts were transferred to Atlas.ti V.8 and coded based on the identified codes and themes. Qualitative and quantitative data were integrated during analysis and in presentation of the results to provide meaning to each data source.

### Ethical *approval*

The study was approved by the Research and Ethics committee of AMREF Health Africa (Protocol number P1212-2022) and a research permit was obtained from the National Commission for Science, Technology and Innovation (NACOSTI) (Permit number NACOSTI/P/22/19335). Approval was also obtained from the Department of Health of Mombasa County Government, and from the administrative leaders in Junda. Respondents received full information about their roles and participation in the study, including potential risks, benefits, their voluntary participation, assurance of privacy, and confidentiality of the information provided. As an indication of their voluntary participation, participants signed a consent form at the beginning of the study after they were satisfied with the information provided to them, and they retained a copy of the signed consent form.

### Preliminary activities: Stakeholder engagement

Two half-day workshops were held with at least 20 stakeholders each from Mombasa County and Kisauni Sub-County in September and November 2023, respectively, to share findings from the previous phase of the study and to identify possible interventions. Stakeholders were selected by the County/Sub-County to ensure diversity and complementarity to the subject matters. These stakeholders represented expertise in public health, Health promotion, Water, Sanitation and Hygiene (WASH), Infection Prevention and Control (IPC), nursing, and health records.

Stakeholders worked in three groups and results from the engagement meeting with county-level stakeholders are presented in Table 1. Notably, the stakeholders proposed focusing on behaviour change and using resourceful individuals within the community. The discussion at the sub-county level also yielded similar results, with stakeholders proposing additional aspects such as analysing the quality of water used for handwashing and ensuring that households have an adequate supply of water.

**Table 1:** Results from group discussions with stakeholders on possible intervention strategies

Group 1	Group 2	Group 3
Behaviour change communication to households (using multiple channels) Education and training to households Use CHPs, chiefs, village elders, religious institutions	Behaviour change communication through multiple channels Encourage community to establish HWF with soap Targeted household visits by CHPs Identify influential persons in the community, i.e. gate keepers Regular monitoring and evaluation	Create awareness through various channels Conduct hand washing demonstration in the community Use of community handwashing champions in creating awareness on handwashing in the community

### Phase 1: Quantitative and qualitative data collection

#### Approach

Seven CHPs from the study area randomly selected 10 willing participants from their community units resulting in a total of 70 potential participants who formed the sampling frame. Eight participants were randomly selected from each of the seven CHPs, resulting in a total sample of 56 participants. A survey was conducted among the selected participants. The survey tool contained questions on sources and uses of water, location of sanitation facilities, types and availability of handwashing facilities (HWFs), availability of soap, and challenges in handwashing with soap. Results from the survey led to the development of an in-depth interview tool to further explore the water, sanitation and hygiene practices in the compounds. Field staff and CHPs again visited the households/compounds to interview the same respondents on their water sources, handwashing facilities and handwashing with soap practices.

#### Findings

A total of 56 and 51 households were interviewed during the survey and in-depth interviews, respectively. A summary of socio-demographic characteristics of the participants, living conditions and water and sanitation facilities are presented in Table 2.

**Table 2:** Summary of socio-demographic characteristics, living conditions, and water and sanitation facilities of respondents

Variable	*n* = 56; Freq (%)
Socio-demographic characteristics	
Gender Male Female	12 (21.4) 44 (78.6)
Marital status Single (never married, widowed, or separated) Married or in partnership	11 (19.6) 45 (80.4)
Highest level of education Primary Secondary or higher None	20 (35.7) 23 (41.1) 13 (23.1)
Main source of income Employment (public or private) Self-employment Casual work None	4 (7.1) 24 (42.9) 14 (25) 14 (25)
Estimate of monthly household income Below 10,000 Kshs Above 10,000 Kshs Prefer not to answer	26 (46.4) 15 (26.8) 15 (26.8)
Number of people in the house	4.9 (1–11) SD 2.01
Living conditions	
Type of house ownership Own Rent Living without pay	16 (28.6) 39 (69.6) 1 (1.8)
Number of rooms in the dwelling unit	2.4 (1–8) SD 1.7
Type of residence Freestanding house (without compound yard) Freestanding house with a private yard Compound with family houses Compound shared with multiple unrelated families A room/house in a block with several houses	7 (12.5) 7 (12.5) 2 (3.6) 14 (25) 26 (46.4)
Shared status of compound* Single HH compound Multiple HH compound/House	10 (17.9) 46 (82.1)
Number of households in compound	6.1 (1–18) SD 4.2
Water and sanitation characteristics	
Primary source of water Tap in the house Tap in the compound Public tap Borehole Protected well Water vendors	7 (12.5) 6 (10.7) 12 (21.4) 6 (10.7) 3 (5.4) 22 (39.3)
Main uses of the primary source of water (multiple response) Drinking and cooking Washing and bathing Flushing toilet Handwashing	45 (80.3) 34 (60.7) 25 (44.6) 16 (28.6)
Secondary source of water Tap (in house, compound or public tap) Borehole Water vendors Protected well None	10 (17.9) 22 (39.3) 17 (30.2) 5 (8.9) 2 (3.5)
Main uses of secondary water source (multiple response) Drinking and cooking Washing and bathing Flushing toilet Handwashing	25 (44.6) 37 (66.1) 35 (62.5) 17 (30.3)
Type of sanitation facility Flush/pour flush toilet to underground tank [septic tank] Flush/pour flush toilet to pit latrine Pit latrine with/without concrete slab (not pour flush) Other (bag/no facility/bush/field, not observed)	28 (50) 6 (10.7) 19 (33.9) 3 (5.4)
Shared sanitation Yes No	39 (69.6) 17 (30.4)

^*^ Single HH compound were where the respondent was the only household in the compound

##### Finding 1: Handwashing facilities were mobile and soap was placed at various locations

The different types of handwashing facilities and handwashing practices are presented in Table 3. Briefly, most (77%) of the handwashing facilities used were basins or buckets. Fifty nine percent of the facilities were mobile facilities (meaning they were not placed at any specific location), 12% were located outside the house and next to the door, and 11% were placed next to the sanitation facility. These results were reflected in the in-depth interviews, with most (*n* = 14) of the mobile facilities being placed outside the house (at various locations), inside the house (*n* = 13), next to the door (*n* = 9), in/near the toilet (*n* = 5) or in other locations such as next to the bathroom.

**Table 3:** Handwashing facilities and practices, and determinants of handwashing with soap

Variables	*n* = 56 Freq (%)	
Type of handwashing facility Basin/bucket with jug/cup/container Basin/bucket (without jug/cup/container) Sink with tap (in the house) Tap (in the house) Tap (in the compound) Bucket/ jerry can with tap	33 (58.9) 10 (17.9) 6 (10.7) 3 (5.4) 1 (1.8) 3 (5.4)	
Location of handwashing facility Mobile facility In the kitchen In the house next to the door Outside the house, next to the door Next to the sanitation facility	33 (58.9) 5 (8.9) 5 (8.9) 7 (12.5) 6 (10.7)	
Category of handwashing facility** Mobile Fixed	43 (76.8) 13 (23.2)	
Soap observed Yes No	22 (39.3) 34 (60.7)	
Use of soap during handwashing Always Sometimes No	12 (21.4) 43 (76.8) 1 (1.8)	
Always handwashing with soap*** Yes No	12 (21.4) 44 (78.6)	
Soap used for handwashing Bar soap Liquid soap (locally made) Liquid soap (commercially produced) Powder soap	12 (21.8) 38 (69.1) 2 (3.6) 3 (5.4)	
Other uses of the soap (multiple response) Only used for handwashing Bathing Cleaning utensils, and all other kitchen uses Laundry and all other washing use	16 (28.6) 8 (14.3) 30 (53.6) 9 (16.1)	
When hands are always washed with soap (multiple response) After defecation After changing the nappy and cleaning/wiping a child’s bottom Before preparing food Before eating Before touching face, mouth, nose, eyes Before breastfeeding the child Before feeding the child (other than breastfeeding) After handling animals After eating After feeding After returning home When visible dirt seen	53 (94.6) 5 (8.9) 23 (41.1) 32 (57.1) 4 (7.1) 4 (7.1) 8 (14.3) 1 (1.8) 33 (58.9) 7 (12.5) 21 (37.5) 35 (62.5)	
Challenges of handwashing with soap (multiple response) Limited access to handwashing facilities Insufficient water supply/lack of water for handwashing Limited availability of hygiene products, e.g. soap or sanitizer Limited access to handwashing education, resulting in a lack of awareness about the importance of hand hygiene None	13 (23.2) 15 (26.8) 13 (23.2) 2 (3.6) 24 (42.9)	
Determinants of handwashing with soap (multivariable logistic regression		
Predictors	*P*	OR (CI)
Soap (ref: soap observed) No soap observed Type of HWF (ref: mobile) Fixed handwashing facility	0.007 0.27	0.09 (0.02–0.54)* 2.29 (0.52–10.01)
Pseudo *R*^*2*^ Wald Chi Number of observations Prob > Chi2	0.24 9.26 56 0.01	

^**^ Basins and buckets, with or without containers, and buckets with a tap were classified as being mobile

^***^ Recreated from the variable on if hands are always washed with soap. Only those who reported that they always washed their hands with soap and water were classified as practising hand washing with soap

Respondents placed these mobile facilities outside their houses because it was convenient to wash hands from outside, because they were shared with their neighbours, or for easy access so that they could be used for other purposes.

*“I also use them* [a basin and jug] *for bathing and washing clothes*” [Female Respondent EM-45-JD]

From the quantitative phase, soap was observed in only 40% of the respondents’ houses/compounds. Twenty-one percent of respondents reported always washing hands with soap and water and the majority (77%) reported that they *sometimes* used soap (Table 3). Similar sentiments were shared during the qualitative phase.

*“I pour the water from the jug inside the basin, then I dip my hands and wash, then I pour the dirty water, refill the basin with clean water and rinse my hands” [Female Respondent* ZI-21-RO]

The quantitative results indicated that over 70% of the respondents reported using liquid soap with the rest using powder soap and bar soap. Similarly, from the qualitative results, most of the respondents (*n* = 42) used liquid soap, powder soap (*n* = 4) and bar soap (*n* = 5). The soap was stored at various locations, depending on other uses of the soap; for example, *“the soap is kept outside for easy access when going to bath…” *[Female Respondent EM-45-JD].

##### *Finding 2: Placement of *handwashing* facilities and availability of soap determined handwashing with soap practices*

Analysis of survey data did not reveal significant associations between socio-economic variables -income and education—and access to handwashing facilities and soap. However, close to 70% of respondents with fixed handwashing facilities had secondary or higher level of education. Chi-square tests showed a significant association between the type of handwashing facility (fixed vs. mobile) and the presence of soap at the facility (χ^2^ (1) = 10.05, *P* < 0.002), as soap was present in 77% of fixed handwashing facilities compared to only 28% of the mobile facilities that had soap. There was also a significant association between the type of handwashing facility (whether mobile or fixed) and the practice of handwashing with soap (χ^2^ (1) = 6.15, *P* = 0.013), as 84% of those who did not always wash hands with soap had a mobile facility. Binary logistic regression confirmed the strength of the association as households where soap was not observed had 92.5% lower odds of handwashing with soap compared to households where soap was observed (*P* = 0.002 CI 0.01–0.39), and households with a fixed handwashing facility had 5.3 times higher odds of practising handwashing with soap compared to households with mobile handwashing facilities (*P* = 0.02 CI 1.32–21.23).

Multivariable logistic regression analysis corroborated these findings, indicating that households where soap was not observed had 90% lower odds of washing hands with soap, compared to households where soap was observed (AOR = 0.09; *P* = 0.01 CI 0.02–0.54) (Table 3). Qualitative data provided contextual insights, suggesting that the predominance of mobile facilities and the use of soap for other household activities made it highly unlikely that soap and the handwashing facilities would be kept at the same place to facilitate handwashing with soap.

### Phase 2: Engagement, education and evaluation

#### Approach

Following the data collection phase, a meeting was held between the CHPs and the research team to review findings and collaboratively determine the focus of subsequent educational activities. Both parties agreed that educational activities should prioritize setting up dedicated handwashing facilities at strategic convenient locations, i.e. in the house for handwashing needs within the house, and in the compound for handwashing when outside the house, e.g. after using the toilet. In response to recommendations from CHPs, an easy-to-use single-leaf instructional guide was developed to serve as a visual tool to support CHPs when delivering educational messages during the visits. One side of the guide outlined the CHPs responsibilities during the visits and the reverse side illustrated various types of locally available handwashing facilities (Appendix 1). CHPs conducted two visits to the households/compounds. The first visit focused on introducing the concept of setting up HWFs at convenient locations. During this visit, the CHPs emphasized the importance of facilities that are specifically designated for handwashing and which are placed at a specific location that facilitates handwashing. The CHPs illustrated the different types of handwashing facilities that were locally available and tasked the respondents to select and commit to setting up a handwashing facility at the compound and household level. The second visit, conducted one week later, served as a follow-up to assess progress and identify any challenges in fulfilling the commitments made by the respondents. Where handwashing facilities had been set up, CHPs encouraged participants to set up a maintenance plan for the handwashing facilities. Where handwashing facilities had not been set up, CHPs probed for challenges/hindrances and continued to encourage the respondents to set up the handwashing facilities and the maintenance plan. Each of these visits lasted for approximately 30–45 min. An in-depth interview guide was then developed for a subsequent follow-up and evaluation visit by the research team. The guide contained questions on the discussions held with CHPs, improvements made, maintenance of the HWFs, learnings gained from the visits, and challenges faced in implementing the recommended changes.

#### Findings

A total of 53 participants were visited by the CHPs. Based on data collected from the CHP monitoring tools, participants committed to various improvements, specifically; introducing an additional handwashing facility next to the toilet at the compound level (*n* = 8), having a designated space for the HWFs (*n* = 6), differentiating the handwashing facilities at the household based on the design, colour or size of the HWFs (*n* = 18), and making improvements in maintenance (e.g. providing soap and water) (*n* = 6). In total, 17 participants opted for buckets/basin and jugs, 15 selected containers (buckets or jerrycans) with taps, 12 selected leaky tins, and 9 preferred to continue using existing taps. Since these activities were happening during the fasting period,^1^ participants promised that they would make the proposed improvements after the fasting month.

The research team interviewed 43 respondents during the follow-up evaluation visit. All participants confirmed that they had been visited by the CHPs and had engaged in discussions concerning handwashing with soap.

##### Finding 1: Participants began setting up handwashing facilities at the compound and household level

Out of the 43 participants, 21(49%) had either established, initiated the set up of, or already had designated handwashing facilities at the compound level. Five participants (12%) maintained the handwashing facilities they had, and 17 (39%) had not undertaken any improvements at the compound level. The facilities that had been set up included improvised jerrycans, 5-L jerrycans that were modified to leaky tins, and buckets. Participants utilized containers available in their households—such as buckets and jerrycans—or purchased additional items such as taps or soap.

“*I improvised a jerry can by cutting off the top …then I labelled it*” [Female Respondent-ER-30-EN]

“*We chose the 5-liter jerry can. We made a hole on the jerry can and placed a bucket beneath* [to collect dirty water during handwashing*]*” [Female Respondent-MIA-50-RM]

Among participants who had not set up HWFs, the main reasons included financial constraints (*n* = 8), being in the process of setting the facilities up (*n* = 5), and lack of cooperation from neighbours (*n* = 4).

At the household level, all the participants reported maintaining existing practices of using mobile facilities (such as basins, buckets and jugs) and sinks. Notably, some participants (*n* = 6) had taken steps to designate and differentiate the mobile handwashing facilities by size or colour of the basins and buckets.

##### Finding 2: Lack of water, soap and reluctance to participate in maintenance were the main challenges

Participants mentioned several challenges in their efforts to set up the handwashing facilities. The unreliable supply of water was the main challenge (*n* = 10) as there had been a few days without regular water supply at the time of the visit. At the time of the follow-up, most of the participants had just set up the handwashing facilities and had only begun maintenance activities such as collecting resources from other households within the compound. However, maintenance of the facilities was mentioned as a challenge—often due to members being away, and others not being willing to participate in the responsibilities.

“*Many times, once the water is finished, there is reluctancy to refill the bucket*” [Male Respondent-MO-62-LA]

Other reported challenges included lack of dedicated soap for handwashing (*n* = 5) and a general reluctance among compound members to wash hands.

As a way forward, participants proposed education to community members (*n* = 12) through various approaches including communal education sessions, media outlets, and individualized household visits. Respondents emphasized the effectiveness of the visual materials used by the CHPs, noting that they illustrated the various types of HWFs that could easily be constructed by community members and recommended the use of such during education activities.

Insights from this follow-up aligned with the recommendations from initial stakeholder engagement meetings, which had similarly proposed education through community dialogue forums. Despite the progress in setting up handwashing facilities, the findings revealed a general lack of knowledge regarding how to wash hands with soap and the importance of washing hands with soap.

### Phase 3: Community dialogue, education and evaluation

#### Approach

**Fig. 2:**
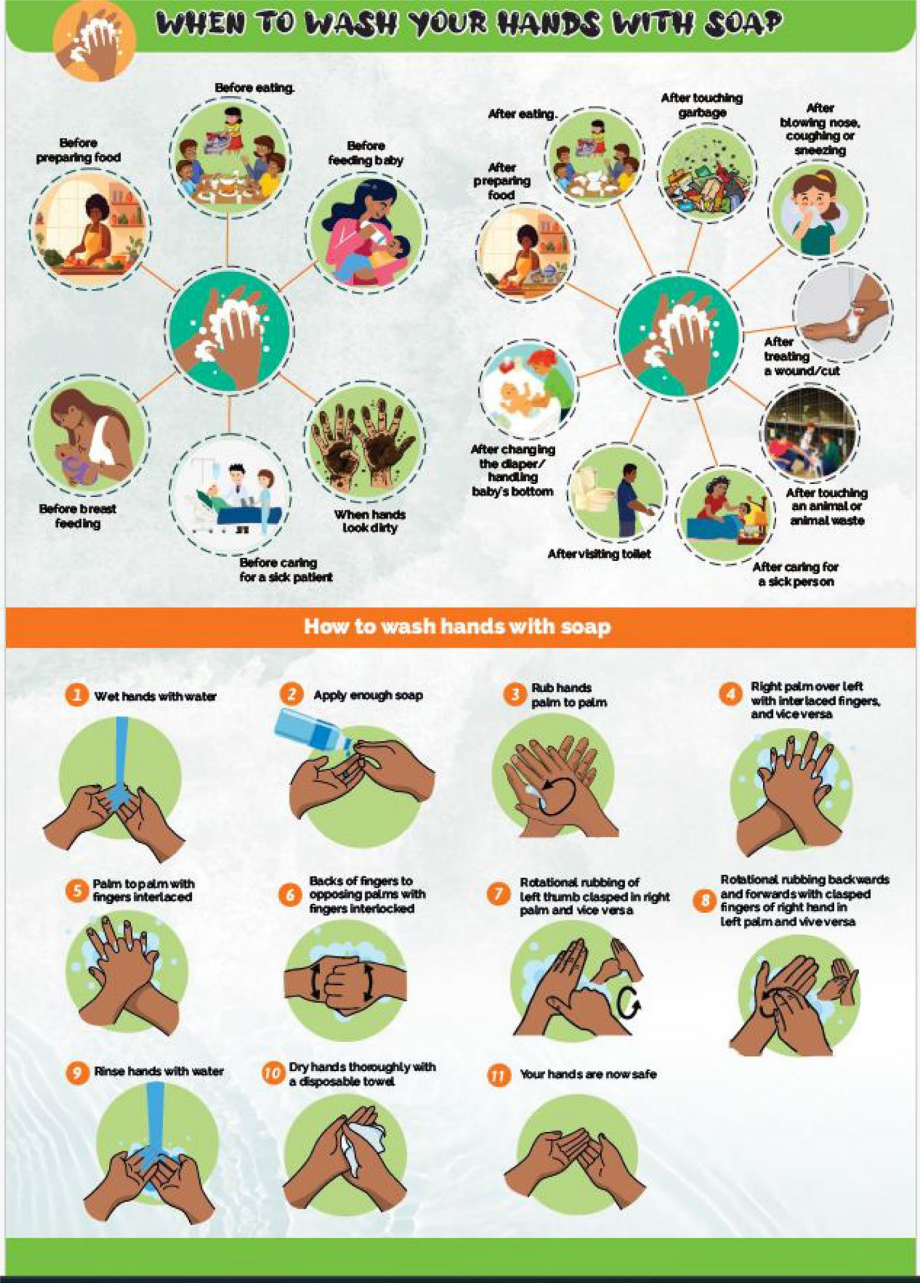
Educational poster used to illustrate when and how to wash hands

Following insights from the follow-up evaluation visit, educational posters were developed to illustrate when to wash hands and the steps of handwashing with soap (Fig. 2).

Study participants were invited to a community dialogue meeting in April 2024 that was attended by other community members, the CHPs, community level leaders, and representatives from the County and Sub-County teams. During the session, participants received practical instructions on how and when to wash hands, and the effects of not washing hands with soap. ‘How to wash hands’ was demonstrated practically using declarative statements that reinforced personal leadership in handwashing, while ‘when to wash hands’ was taught using the posters. Key times for handwashing were specified as before and after handling food, eating/feeding, and handling a sick patient; and after sneezing/blowing nose, defecating, handling faecal matter, touching garbage, and touching wounds. The steps of handwashing included wetting hands, applying soap, rubbing and scrubbing all areas of the hands, rinsing, and drying.

CHPs and field teams visited study participants the day after the community engagement meeting to monitor progress, identify challenges, and reinforce handwashing education. An in-depth interview tool was used to assess improvements in the installation or designation of handwashing facilities at both household and compound levels, as well as maintenance of the handwashing facilities. Where participants had not made improvements, CHPs encouraged them to make the necessary adjustments, especially in developing a maintenance plan. After the discussions, field staff and the CHPs provided posters to the participants, practically guiding them on the contents of the poster (when and how to wash hands). Participants were asked to place the poster at the most convenient location. They were also informed that a follow-up visit would be conducted to gather feedback on their experience with the posters.

Approximately 3 weeks later, CHPs and the field staff returned for a follow-up and evaluation visit. An interview guide was developed and used by the field staff as a guide in the discussions with households. The guide had questions to assess where the posters were placed and how they were used, what participants liked and disliked about the posters and the visits, lessons learnt and improvements made, and proposed areas for further improvement. Participants were also invited for the last community dialogue meeting to consolidate learnings and share experiences. Each of these visits lasted approximately 30–45 min depending on where the respondents were with setting up their handwashing facilities.

##### Finding 1: Continued setting up of handwashing facilities resulting in increased handwashing practices

A total of 48 households were reached during the visit to deliver the posters. From this visit, 10 participants, who had not set up their handwashing facilities during the previous visit, had set their facilities at the compound level. All 10 participants reported making use of mobile facilities for handwashing (except for 5 who had sinks within their houses) at the household level. One participant who had a sink at the household level introduced homemade disinfectant for use. Just like in the previous visit, the participants reported differentiating their handwashing facilities at the household level by size, colour or where they were kept.

In addition to the previously reported benefits, five participants noted that there was an increase in handwashing among family or compound members.

“*When they* [Tenants] *come out of the toilet, they wash their hands. Even children wash their hands*” [Female Respondent-FB-27-EN].

The challenges of inadequate finances to purchase handwashing facilities, lack of water, and lack of cooperation as reported previously were still reported during this visit. Other challenges included lack of soap (*n* = 3). Participants still recommended additional education for other members in the compound and in the community.

##### Finding 2: Reported increased knowledge of handwashing practices from educational materials

A total of 46 participants were interviewed during the follow-up evaluation visit. Most (*n* = 21) of the posters had been positioned next to or near the toilet, while 9 had been placed next to or near the HWF. Other areas where the posters had been placed included 6 at the compound entrance, 4 at the main participants door, and 2 within the corridor. Four posters had not yet been placed because the participants were away, but they promised to place them immediately. Notably, 4 of those that were placed near the HWFs were in households where the participants used sinks, and the rest were near the HWFs that were often near/next to the toilets within the compound. All the participants affirmed that the selected locations were the most convenient as the posters could be seen by most of the other compound and household members.

“*I placed it on the door because people can see it directly*” [Female Respondent-EDB-42-JD]

However, 4 posters were missing because they had fallen off or had been plucked out by other compound members or by unknown people.

“*I had travelled and when I came back, I didn’t find it* [the poster]*. When I asked where it was, one* *[neighbour’s] child said that they removed it and stuck it elsewhere*” [Female Respondent ZB-01-PM]

Participants reported that the posters clearly illustrated when and how to wash hands, and that the message was easily understood by other users, including children.

“*Even this child *knows* that they wash hands here because they see others do it*” [Male Respondent HN-06-PM]

Six compounds reported having come up with ways of working together with other compound members in maintenance of the HWF, which included using a duty rota (*n* = 5) or voluntary participation of other compound members. Participants still reported the previous challenges with additional challenges of theft of handwashing facilities (*n* = 2).

“*At first I was *the* only one* [involved in maintenance] *but there is now a duty rota and my fellow tenants help me.”* [Female Respondent-ER-30-EN]

“*There is no specific person who is responsible for refilling, if anyone sees the facility empty, they refill it*” [Male Respondent SA-23-RO]

All the participants noted that their knowledge and practice of handwashing with soap had improved due to the frequent educational visits.

### Phase 4: Quantitative evaluation

A second community dialogue meeting was convened with study participants in May 2024 with an objective of providing a platform for participants to share experiences, insights, and lessons learned throughout the intervention period. Participants unanimously reported improvements in their knowledge and handwashing practices, attributing these changes to the education and engagement activities. They collectively recommended that the research team conduct a follow-up evaluation one month later to assess sustainability and progress of the improvements initiated by the process.

Following the commitments expressed by participants, a follow-up survey was conducted one month later to assess the sustainability of handwashing practices. Field staff and CHPs conducted household visits to the same households to assess whether participants maintained the recommended practices. A survey tool was developed to capture changes that had been made, including water sources and uses, availability and location of handwashing facilities, availability and use of soap, handwashing techniques, knowledge of handwashing moments, improvements made since the interventions started, and challenges in implementing the interventions. This follow-up assessment provided insights into the extent of behavioural adoption and the practical challenges faced by households in sustaining the improvements.

#### Findings: Increased availability of handwashing facilities and soap, and improved handwashing with soap practices

A total of 44 participants were surveyed during the follow-up survey and these results are presented in Table 4. Handwashing facilities were observed in most (77%) compounds, and these were located at or near the toilet for handwashing after toilet use. Twenty-five percent of these facilities were buckets/jerrycans with taps and 20% were customized containers. Most (70%) of the handwashing facilities used in the house were mobile facilities such as basins placed in various locations within the house. Soap was observed in 64% of the facilities located near the toilet and in 60% of the facilities in the house. Regarding handwashing practices, 64% reported always using soap for handwashing with 97% of the soap being liquid soap.

**Table 4:** Handwashing facilities and practices one month after educational visits

Description	*n* = 44 (%)
Type of compound handwashing facility after toilet use (HWF1) # Basin/bucket and jug Bucket/jerrycan with tap Customized container Leaky tin/tippy tap Sink Others (tap and none)	14 (31.8) 11 (25) 9 (20.4) 5 (11.3) 3 (6.8) 2 (4.5)
Location of HWF 1 Outside the house, next to the door Inside/next to the sanitation facility Other (outside the compound, in living room, not in compound)	7 (15.9) 34 (77.3) 3 (6.6)
Type of household HWF (HWF 2) # Basin/bucket and jug Sink Bucket/jerrycan with tap Tap Others (leaky tin/tippy tap)	31 (70.4) 4 (9.1) 3 (6.8) 2 (4.6) 4 (9.1)
Location of HWF 2 In the kitchen/near the kitchen area In the living room In the house next to the door Outside the house, next to the door N/A (i.e. they use HWF 1)	10 (22.7) 18 (40.9) 10 (22.7) 3 (6.8) 3 (6.8)
Soap observed near HWF 1 Yes No N/A	28 (63.6) 14 (31.8) 2 (4.5)
Soap observed near HWF 2 Yes No N/A	26 (59.1) 15 (34.1) 3 (6.8)
Knowledge and practice of handwashing with soap	
How hands are washed (multiple response) Using soap and water in a basin Using soap and running water Dipping hands in a basin of water Washing hands with running water	20 (45.4) 38 (86.3) 3 (6.8) 6 (13.6)
Use of soap during handwashing Always Sometimes No	28 (63.6) 16 (36.4) –
Always handwashing with soap* Yes No	28 (63.6) 16 (36.4)
Soap used for handwashing Liquid soap (locally made) Powder soap	43 (97.7) 1 (2.3)
Other uses of the soap (multiple response) Only used for handwashing Bathing Cleaning utensils, and all other kitchen uses Laundry and all other washing use	9 (20.4) 4 (9.1) 33 (75) 18 (41)
When hands are always washed with soap (multiple response) After defecation After changing the nappy and cleaning/wiping a child’s bottom Before preparing food Before eating Before breastfeeding the child Before feeding the child (other than breastfeeding) After handling animals After eating After feeding After returning home When visible dirt seen	39 (88.6) 8 (18.8) 19 (43.2) 25 (56.8) 1 (2.2) 2 (4.55) 1 (2.2) 17 (38.6) 6 (13.6) 25 (56.8) 22 (50)
When hands are washed without soap Before eating Before breastfeeding the child Before feeding the child (other than breastfeeding) After eating	4 (9.1) 2 (4.5) 1 (2.2) 3 (6.8)
Improvements made to the HWF made since the start of visits Yes No	41 (93.2) 3 (6.8)
Specific improvements made (*n* = 41) Designated a HWF at the toilet (including placing poster) Designated HWF in the household Added a soap container/dispenser Improved access to the handwashing facility (identified a place, elevated the HWF Improved signage for handwashing instructions Increased frequency of cleaning and maintenance of the HWF Added another HWF Others (education to the family and compound), added paper towels, set up a tap in the compound)	21 (51) 16 (39) 16 (39) 6 (14.6) 3 (7.3) 10 (24.3) 7 (17) 6 (14.6)
Improvements made since the last visit** Designated a HWF at the toilet Added a soap container/dispenser Improved accessibility and use of handwashing facility Increased frequency of cleaning and maintenance of the HWF Shared information about HW with others	1 (2.2) 6 (13.6) 3 (6.8) 7 (15.9) 6 (13.6)
Other improvements after the last visit** Improved hygiene practices Continued maintenance activities None Continued education Others (learnt how to make liquid soap, shared soap)	4 (9) 3 (6.8) 22 (50) 11 (25) 4 (9)
Factors that mostly influenced positive change (*n* = 36) Visits, training and practical demonstration Improved hygiene and health outcomes Posters Separation of HWFs and availability of soap	13 (36) 11 (30.5) 4 (11.1) 8 (22.2)
Motive for continued HWWS throughout the study period Improved health outcomes Regular education visits from the CHPs Regular education visits from the research team Supply of soap from the team	28 (63.6) 10 (22.7) 14 (31.8) 13 (29.5)
Challenges experienced Yes None	32 (72.7) 12 (27.3)
Challenges (multiple response) Insufficient water supply Lack of resources, thus limited availability of hygiene products such as soap Lack of cooperation from household and compound members Mismanagement challenges (theft of HWF, ‘water wastage by children)	12 (37.5) 18 (56.2) 15 (46.8) 8 (25)

^#^ HWF 1 was the handwashing facility shared by households within a compound, and HWF 2 is the handwashing facility for use within a household

^*^Recreated from the variable on if hands are always washed with soap. Only those who reported that they always washed their hands with soap and water were classified as practising hand washing with soap

^**^This refers to the final educational visit (phase 3)

Overall, 93% had made improvements since the beginning of the study; these improvements being installing handwashing facilities at or near toilets (51%), designating household level handwashing facilities (39%), introducing soap at handwashing facilities (39%), and increasing the number and maintenance of the handwashing facilities (24%) (Table 4).

Participants were asked to identify factors that influenced these positive changes during the study period. Overall, 36% mentioned that the household visits, training and practical demonstrations were the most impactful, 30% mentioned their own desire for improved hygiene and health outcomes, and 22% highlighted the availability of handwashing facilities and soap.

In comparing these findings to the findings before the beginning of the educational visits, there was a substantial increase in the availability of handwashing facilities—from 10% who had mobile facilities near the toilet to 77% with facilities located next to or near the toilet. There was an increase in the types of facilities, ranging from basins/buckets (32%) to customized containers (buckets, jerrycans) with or without taps (45%), and leaky tins/tippy taps (11%). Soap availability increased from approximately 40% before beginning of intervention activities to 64% near toilets and 60% within households.

There was a significant increase in those who reported always using soap from 21% at the beginning of the study to 64% at the end of the intervention activities (McNemar's *X*^*2*^(1) = 12.46; *P* = 0.00). All the participants confirmed that their knowledge and practice of handwashing with soap had improved as a result of the frequent educational visits.

“*Your visits *have* made me so happy… I didn’t know how to wash hands the correct way but now I know*”. [Female Respondent RM-32-EN]

A summary of these practices at the beginning and the end of the intervention activities is presented in Tables 3 and 4.

## Discussion

This paper details the development of a handwashing intervention implemented in low-income settlements of Mombasa. The intervention was guided by previously identified barriers to handwashing with soap and aimed to address these through targeted strategies that enhanced both the physical capability for handwashing and the knowledge and skills required to sustain the practice. Key activities during the development phase included stakeholder engagement and household-level education to promote behaviour change. Strategies were introduced sequentially and involved collaboration with CHPs to encourage setting up and designation of handwashing facilities within compounds and households. Practical demonstrations and the use of education materials were employed to reinforce knowledge on effective handwashing with soap. By the end of the intervention, over 70% of compounds had set up handwashing facilities near their shared sanitation facilities and reported handwashing with soap significantly increased from 21 to 64% by the end of the intervention activities. A notable challenge encountered was the lack of consistent cooperation from other compound members in maintenance of the handwashing facilities which affected sustainability at the compound level.

A core principle guiding the development of this intervention was the engagement of diverse stakeholders including County officials, administrative and community leaders, CHPs, and public health authorities in both the design and implementation phases. Such engagement often leads to fostering social cohesion, enhancing community ownership, and increasing the likelihood of intervention uptake and sustainability [17, 18, 33, 34]. In this study, engagement of stakeholders also encouraged broader community participation in intervention activities. CHPs played a central role in household-level engagement, serving as key facilitators of the intervention. They are an integral part of the health system, and several studies have detailed the experiences of CHPs in delivering and implementing community interventions [35, 36]. The intervention development process involved collaborative efforts between the research team and CHPs, whilst having the CHPs visit the households as part of their routine work as has been done in other approaches [26]. This partnership fostered mutual learning and helped address capacity gaps within the teams. Overall, community members expressed satisfaction with the activities, an indication that stakeholder engagement also contributes to acceptance and success of behaviour change interventions.

The intervention was designed to encourage households to make handwashing-related improvements using their own resources, rather than relying on external provision of facilities or soap. This approach was guided by the Behaviour Change Wheel (BCW) framework, specifically the strategies of enablement and training [37]. Through continuous education, participants set up handwashing facilities to facilitate handwashing. The observed improvements suggest that households are capable of enhancing their hygiene conditions when equipped with the appropriate knowledge and skills. This finding aligns with existing literature on empowerment, which emphasizes that individuals, households and communities can be mobilized to take action when they are actively involved in interventions and provided with information that supports informed decision-making [38]. Such an approach is relevant for local level decision-makers and stakeholders operating in resource-constrained settings where direct provision of handwashing infrastructure may not be feasible. In this study nonetheless, there still were other costs related to supporting CHPs, payment for venues, and production of education materials. To support broader adoption, it may be necessary to estimate all costs associated with such interventions as this information can inform planning, budgeting and policy uptake. Notably, participants provided soap as evidenced by the presence of soap next to or near the handwashing facilities. Studies have pointed to the importance of having a handwashing facility to improve handwashing with soap [39], and our results support this finding, as the presence of the handwashing facility encouraged provision of soap at the fixed facility, thereby enhancing handwashing with soap practices.

Visual materials such as posters were incorporated into educational activities to reinforce hygiene messaging. The influence of lack of knowledge was evident from results from the survey, and was also highlighted in an earlier study as a barrier to effective handwashing signifying the need to address this by increasing knowledge on handwashing with soap in Mombasa [40]. Hygiene education interventions have been implemented through communal approaches such as mass media campaigns [41] or through one-on-one approaches in individual settings [8, 42]. Evidence suggests that educational strategies generally promote the adoption of handwashing behaviour; however, such interventions need to be simplified for better uptake and effectiveness [42]. Our approach consisted of practical demonstrations during community dialogue meetings and individualized education sessions. We adopted the community level approach to leverage the influence of community leaders who were identified as key change agents. Individualized education sessions ensured that all participants are reached, which was particularly important given the low levels of formal education among participants. Both approaches used simplified approaches including demonstration sessions, and these facilitated comprehension and uptake of the messages among participants.

One of the main challenges identified was the maintenance of shared handwashing facilities. To address this, households adopted various strategies such as a duty rota and pooling resources together, strategies that have also been documented in other WASH studies [43–46]. These strategies underscore the critical role of social capital in low-income settings, where WASH services are commonly shared among multiple households. This challenge suggests that in such spaces where WASH facilities are shared, a common mode of operation should be defined as early as possible to ensure sustainability of the interventions, and without which other individuals may be less inclined to participate in maintenance of shared WASH facilities [47]. Proactive planning and community-led governance mechanisms are therefore essential to foster long-term maintenance and ownership of shared WASH facilities in low-income areas.

A key limitation of this study lies in its adaptive design, wherein the intervention strategies were not pre-defined, but instead evolved and were refined through the development process. As a result, certain components—such as the end of intervention survey—were incorporated in later stages of the process, which may have affected the consistency of data collection tools. Additionally, the study employed a small sample size consistent with the methodological guidance of the TIPs approach [32]. The small sample size and the lack of a pre-defined approach limited complex analysis of the data. Nonetheless, the study’s strength lies in its extended testing period which enabled incremental introduction of intervention activities and provided participants with sufficient time to internalize and adopt handwashing practices. The iterative nature of the process also allowed for continuous refinement based on participant feedback, enhancing contextual relevance and potential sustainability of the intervention.

## Conclusion and implications for practice

The intervention development process detailed in this study underscores the importance of stakeholder engagement throughout the development of an intervention. The study demonstrates that communities can be empowered to take their own initiatives in setting up handwashing facilities to improve handwashing with soap. In low-income areas, where WASH facilities are shared, setting up handwashing facilities in locations where handwashing with soap can be done at critical times such as after toilet use is crucial and necessary. Educational interventions play a pivotal role in promoting handwashing with soap, but these education initiatives must be tailored to the unique contexts of the communities involved to maximize their impact. Our development process signifies that education and enablement activities can be integrated in public health interventions and programmes, and CHPs can be included in delivering these interventions as they enhance the effectiveness of health interventions through localized knowledge and trust. This process led to the development of an intervention that is mainly an enablement of the communities, and that focuses on education through community dialogue meetings and household level education activities using simplified approaches such as demonstrations and illustrative materials. Through this approach and based on the proposed strategies from the BCW framework, participants made improvements in their living spaces (a form of environmental restructuring) by creating an enabling environment for handwashing with soap. This integrated approach could serve as a model for future public health initiatives aimed at improving hygiene practices in similar settings. Lastly, establishing clear guidelines for the maintenance of communal handwashing facilities is crucial to ensure the long-term success and sustainability of such interventions in compounds where WASH services are shared.

## Supplementary Information

Supplementary material 1.

## Data Availability

Data are provided within the manuscript or supplementary information files.

## Footnotes

1 These activities were going on during the holy month of Ramadhan.
