# Supplementary material for: Development of a handwashing with soap intervention in low-income settlements of Mombasa, Kenya
Source: Trop Med Health. 2025 Nov 18;53:164. doi: 10.1186/s41182-025-00842-1 (PMC12625283; doi:10.1186/s41182-025-00842-1)

Handwashing with soap in Machafuko village

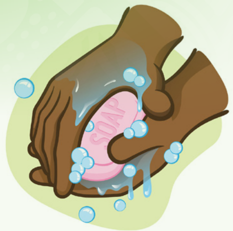

CHPs visit plan to the households

Main message: It is important to wash hands with soap as often as possible since hands are carriers of germs

Week 1

Main message and tasks

Key message

Handwashing is possible if there is a facility that is **designated** for handwashing and placed at **specific** locations.

Assignment for the households

1. Households in compounds/compound houses should come up with/identify a suitable handwashing facility that will specifically be used for handwashing after toilet use and at any other time while outside. This facility should be placed next to or near the sanitation facility.

2. Target households should be encouraged to come up with/identify a suitable handwashing facility that will specifically be used for handwashing within the house.

Households should be encouraged to determine how the handwashing facility will stand out as being specific to handwashing.

Target recipients

1. Compound/structure owners, caretakers or 'senior tenants'.
2. Our household respondents

Task

Show the participant the various types of handwashing facilities and encourage them to select a handwashing facility they can adopt.

At the end of the visit:

CHPs to complete the household visit form and have the respondent sign as a show of commitment.

Week 2

Main message and tasks

Key message

For sustained handwashing practices, handwashing facilities should always be functional (i.e. maintained).

Assignment for the households

1. Ask the respondents to design a maintenance structure for the handwashing facility. For the handwashing facility that is placed near the sanitation facility, households can be encouraged to align it with the duty rota that is used for cleaning their shared sanitation facility. If such a rota does not exist, households should be encouraged to come up with a maintenance plan.

Target recipients

1. Compound/structure owners, caretakers or 'senior tenants' (for the shared handwashing facility)
2. Household respondents (for the household level handwashing facility)

Task

Follow up with the target recipients to identify if the handwashing facilities have been set up. If the handwashing facilities have not been set up, they should be encouraged to honor their commitment and set up the handwashing facility.

At the end of the visit:

Complete the household visit form and have the respondent sign to indicate compliance with the agreed commitment.

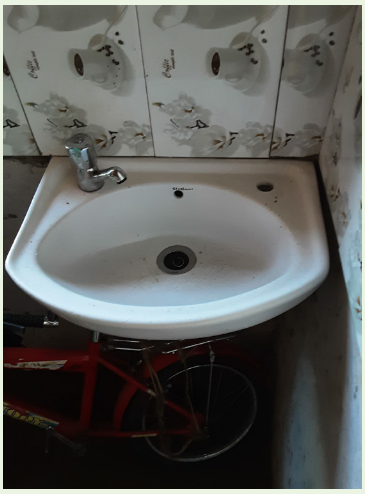

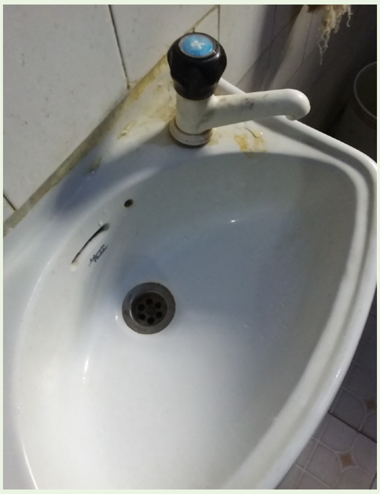

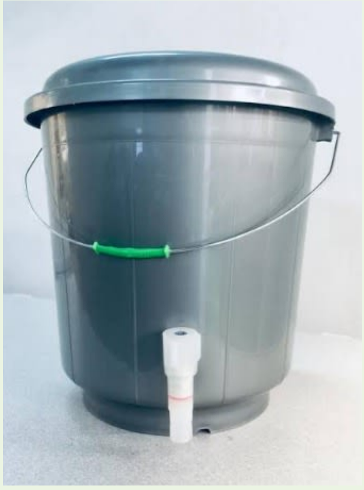

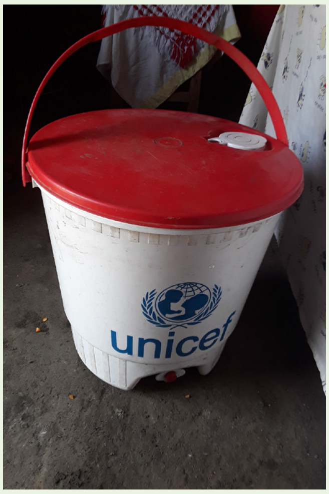
unicef

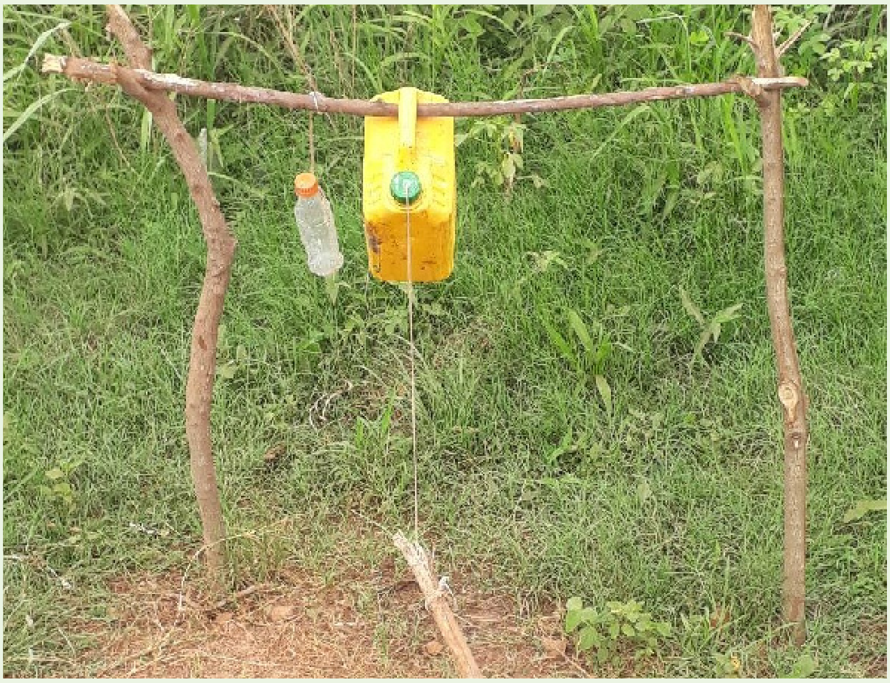

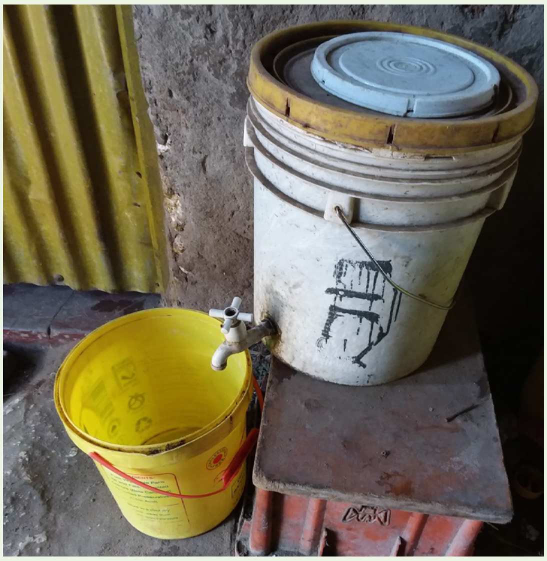

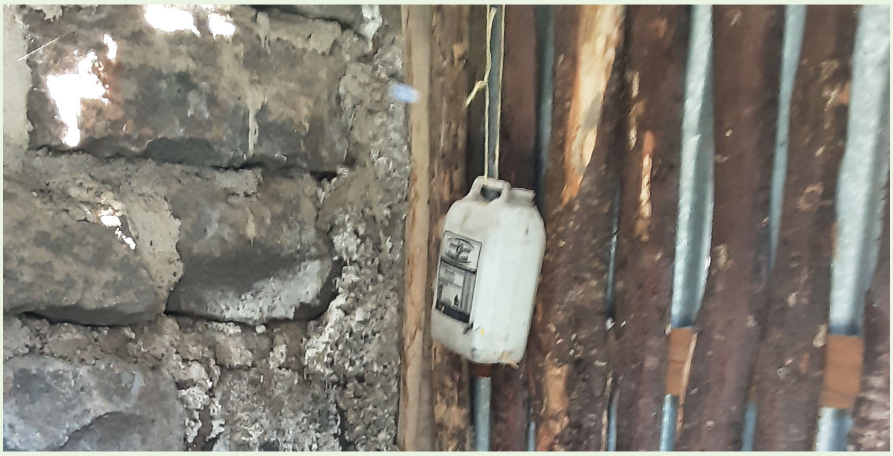

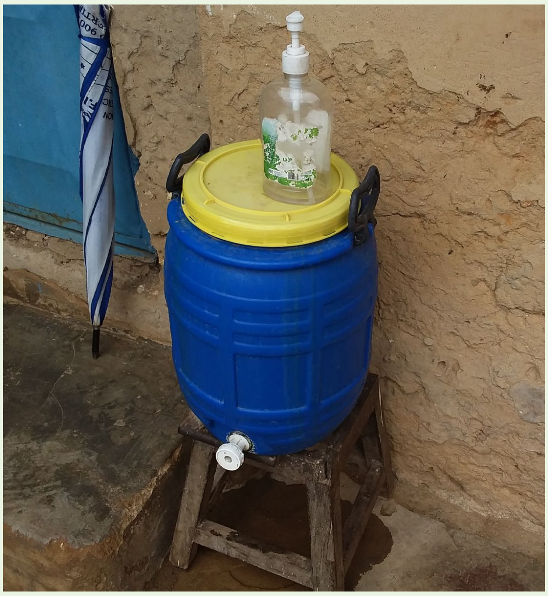

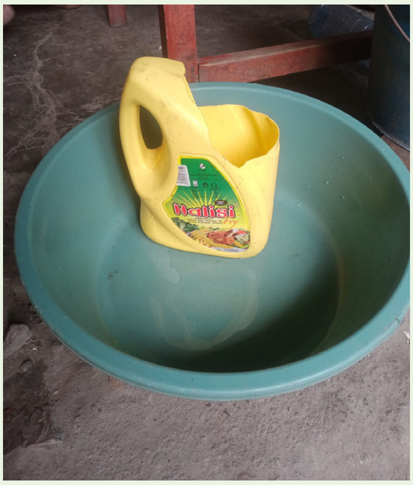
Halisi

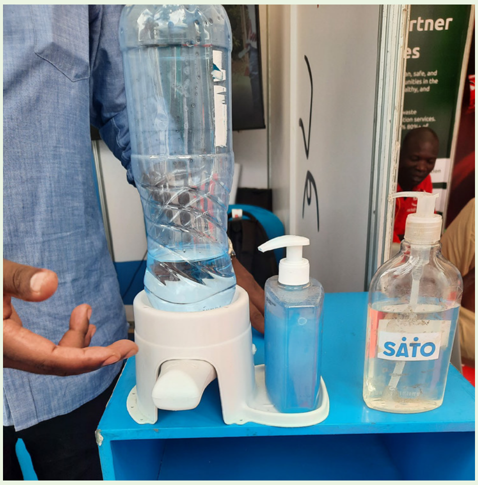
SATO

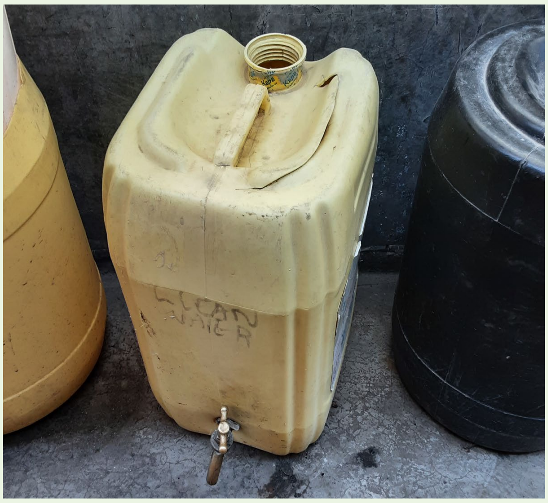

Supplement: Supplementary file 1 — Supplementary material 1. [file 41182_2025_842_MOESM1_ESM.pdf]
